# Databases for Research and Development

**DOI:** 10.3201/eid2510.181411

**Published:** 2019-10

**Authors:** Michael G. Head

**Affiliations:** University of Southampton, Hampshire, UK

**Keywords:** investment, funding, databases, research, development, R&D

I welcome the findings of Mehand et al. in putting together a methodology that can prioritize emerging infectious diseases in need of research and development (*1*). These approaches are vital in establishing how global research funders and research institutions can best contribute to establishing a knowledge base around what diseases to address and how.

There is also a distinct need to understand ongoing research portfolios at international and national levels. The data emerging from these projects can provide further knowledge and impact in health policy and inform further research priorities.

Our ongoing project involves the Research Investments in Global Health (ResIn) study. ResIn has described research portfolios for cancer and infectious disease research in the United Kingdom (*2*,*3*). Internationally, the study has covered investments into global pneumonia research (*4*) and malaria research across Africa (*5*). Findings have examined, for example, the burden of disease alongside levels of investment, as well as providing informed comment on research gaps. ResIn also considers how best to implement findings from a research database into health policy and practice, and has presented results and sought opinion from meetings with key stakeholders, including the World Health Organization (WHO), European Commission, and Wellcome Trust.

I encourage WHO and other stakeholders to consider an open-access global research investments portfolio for all areas of health, using open datasets to describe spending on research alongside other areas, such as burden of disease. Alongside the WHO R&D Blueprint (https://www.who.int/blueprint), this resource can support decision-making around research knowledge and innovation.

## References

[1] Mehand MS, Millett P, Al-Shorbaji F, Roth C, Kieny MP, Murgue B. World Health Organization methodology to prioritize emerging infectious diseases in need of research and development. Emerg Infect Dis. 2018;24:24. 10.3201/eid2409.171427 30124424 PMC6106429

[2] Maruthappu M, Head MG, Zhou CD, Gilbert BJ, El-Harasis MA, Raine R, et al. Investments in cancer research awarded to UK institutions and the global burden of cancer 2000-2013: a systematic analysis. BMJ Open. 2017;7:e013936. 10.1136/bmjopen-2016-013936 28428185 PMC5775472

[3] Head MG, Fitchett JR, Nageshwaran V, Kumari N, Hayward A, Atun R. Research investments in global health: a systematic analysis of UK infectious disease research funding and global health metrics, 1997–2013. EBioMedicine. 2015;3:180–90. 10.1016/j.ebiom.2015.12.016 26870829 PMC4739409

[4] Brown RJ, Head MG. Sizing up pneumonia research. Southampton (United Kingdom): University of Southampton; 2018 [cited 2019 Aug 3]. https://figshare.com/articles/Sizing_Up_Pneumonia_Investment/6143060

[5] Head MG, Goss S, Gelister Y, Alegana V, Brown RJ, Clarke SC, et al. Global funding trends for malaria research in sub-Saharan Africa: a systematic analysis. Lancet Glob Health. 2017;5:e772–81. 10.1016/S2214-109X(17)30245-0 28668230 PMC5567191

